# A simple and cheap home built laparoscopic trainer

**DOI:** 10.4103/0972-9941.43095

**Published:** 2008

**Authors:** Robert Dennis

**Affiliations:** Department of General Surgery, Luton and Dunstable Hospital, Lewsey Road, Luton LU4 0DZ, United Kingdom

**Figure 1:**
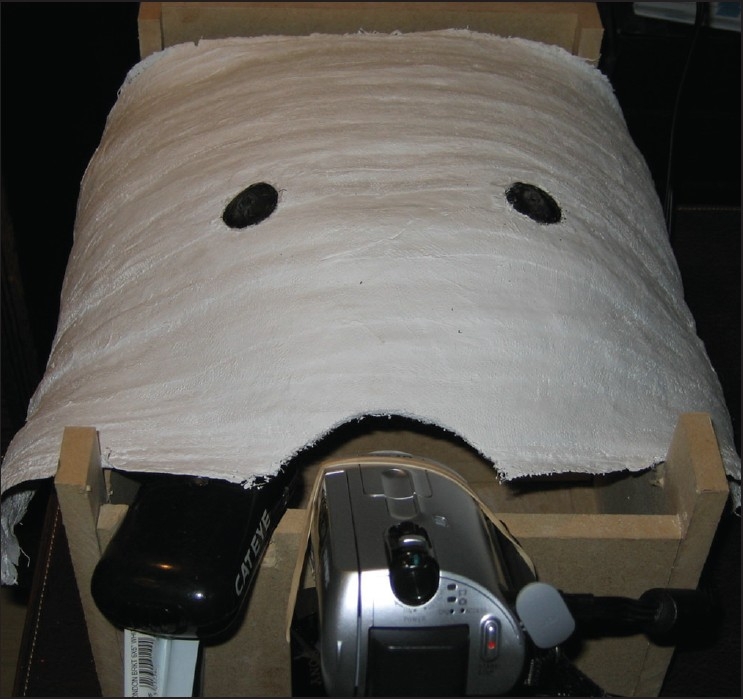
Laparoscopic trainer

The benefits of laparoscopic trainers in the improvement of laparoscopic surgical skills are well established. Unfortunately simulators and trainers are expensive and sparsely available in surgical departments. Home built laparoscopic trainers on much smaller budgets have been described.[1] The base and four vertical walls of a trainer can be constructed with the most basic of woodwork skills. However constructing a suitable top is more challenging. There a few features the top should have. Firstly, its overall structure needs to be sufficiently rigid and robust to act as a fixed fulcrum point for the instruments. Secondly, the ports sites themselves must allow sufficient freedom of movement for manipulation of the instruments. An additional benefit would be a top which modelled the contours of an abdomen. It also needs to be cheap and easy to construct. Such a top can be made from five to six layers of plaster of Paris moulded over an abdomen protected with petroleum jelly. This gives a rigid and sufficiently robust structure. Port sites can be positioned to the user's choice. To allow flexibility at the fulcrum points of the abdominal wall, 3 cm rubber grommets from a car parts shop are glued into holes made into the plaster cast. The camera system is provided by a freely available camcorder and the light source of a bike light. These are supported by shelf brackets commonly available in hardware shops [Figure 1]. Depending on the type of camcorder this trainer could be built for less than £150.
